# Association between fire-safe cigarette legislation and residential fire deaths in the United States

**DOI:** 10.1186/2197-1714-1-10

**Published:** 2014-04-24

**Authors:** Rebecca K Yau, Stephen W Marshall

**Affiliations:** Injury Prevention Research Center, University of North Carolina at Chapel Hill, 137 East Franklin Street, Suite 500, Chapel Hill, NC 27599-7505 USA

**Keywords:** Fire-related mortality, Fire-safe cigarette, Home injury

## Abstract

**Background:**

Cigarettes and other tobacco-related smoking products have traditionally been a major ignition source for residential fire deaths. In the United States, all 50 states and the District of Columbia have passed laws requiring that cigarettes self-extinguish if they are not being smoked (so-called fire-safe cigarette laws). The purpose of this study was to quantify the association between state-level implementation of fire-safe cigarette legislation and the rate of residential fire death.

**Methods:**

Poisson regression was used to analyze state-years data. Main intervention: Implementation dates for fire-safe cigarette legislation in each state. Outcome: Residential fire mortality rate.

**Results:**

Implementation of fire-safe cigarette legislation was associated with a 19% reduction in overall residential fire mortality rates, adjusted for demographic differences between states (rate ratio = 0.81, 95% confidence interval: 0.79, 0.84). This is approximately similar to the estimated proportion of residential fire deaths in which smoking materials are an ignition source (23%). Legislation implementation was associated with a protective effect for every age, sex, race, and ethnicity strata that we examined. State-specific residential fire mortality death rates decreased (defined as a drop of at least 5%) in 32 states after fire-safe cigarette legislation was implemented. In 12 states there was either less than a 5% decrease or an increase, and seven states had insufficient deaths to evaluate state-level changes.

**Conclusions:**

Implementation of fire-safe cigarette is associated with reductions in residential fire mortality rates.

## Background

Deaths from residential fires accounted for 1.9% of all unintentional injury deaths in the United States (US) in 2010, with a mortality rate of 7.3 per 1,000,000 person-years (National Center for Injury Prevention and Control 2013). Approximately 23% of these deaths in 2010 (n = 540) were attributed to ignition of “smoking materials”, typically defined as tobacco products that can be lit, such as cigarettes (Hall Jr. 2013).

Like many injuries, residential fires and resulting deaths reflect a complex interplay of numerous factors, including ignition sources, structural feature of the dwelling, alarm and sprinkler systems, behavior responses, alcohol and substance use, and mobility issues in exiting a burning structure (Baker et al. 1992; Marshall et al. 1998; Runyan et al. 1992). Unintentional residential fire death due to ignition of smoking materials is a source of injury mortality that is highly preventable, since cigarettes have the capacity to smolder and ignite flammable materials in the home (such as bedding) many hours after they have been carelessly discarded. One intervention to address this issue, now widely adopted in the US, is a design standard for cigarettes. This standard requires that cigarettes self-extinguish when they are not being actively smoked. Legislative efforts requiring this design standard at the local point of sale have been highly successful. By 2011, all fifty states and the District of Columbia (DC) had implemented legislation requiring all cigarettes sold locally meet the “fire-safe” standard (Hall Jr. 2013).

One method for making a cigarette fire-safe is by including two to three thin bands of paper in the column of the cigarette that are less porous than standard cigarette paper. To qualify as fire-safe, cigarettes must meet a preset testing standard benchmarked to ASTM E2187, which is a “test method [that] enables comparison of the relative ignition strength of different cigarette designs” (ASTM International 2013). The ASTM E2187 protocol consists of lighting a cigarette, then placing it on filter paper and determining whether or not the cigarette burns the full length of the tobacco column. To test a given cigarette, this protocol is repeated 40 times per cigarette, and the proportion of cigarettes that burn the entire length is the test result. Cigarettes are considered fire-safe if 25% or fewer burn the entire length (Gann 2007).

The earliest efforts to pass fire safe cigarettes (FSC) legislation began in the 1920s, and efforts were renewed in the 1970s (Barillo et al. 2000; Brigham and McGuire 1995). By 1984, the Cigarette Safety Act was passed in Congress, and “the three-year study mandated by this act concluded that it was technically feasible to produce a cigarette with a reduced propensity to start fires” (Brigham and McGuire 1995). Ten years after the Cigarette Safety Act was passed, the Fire-Safe Cigarette Act of 1994 was created to establish “a standard by which cigarettes could be regulated with respect to their propensity to start fires” (Brigham and McGuire 1995). This bill was stalled after the midterm elections in 1994, but was reintroduced in 1999 as the Fire-Safe Cigarette Act of 1999. The 1999 act failed to be enacted at the federal level. However, state-level FSC legislation was successfully passed. The first FSC legislation was passed in New York in 2000 (Rose 2007) and the final state (Wyoming) passed FSC legislation in 2010 (Coalition for Fire-Safe Cigarettes 2011).

The purpose of this study was to assess the association between state-level implementation of FSC legislation and the residential fire death rate. Since FSC legislation has been widely implemented in the US, it is appropriate to evaluate whether the passage of FSC legislation is associated with a reduction in home fire death rates.

## Methods

### Outcome

The outcome of interest was the annual rate of unintentional residential fire deaths in each state and DC. Data for all deaths for all 50 states and DC from 2000–2010 were obtained from the National Center for Health Statistics (NCHS) Mortality-State Only data, as compiled from data provided by the 57 vital statistics jurisdictions through the Vital Statistics Cooperative program.

A death was considered unintentional and fire-related if one of the causes of death was exposure to smoke, fire, and flames [International Statistical Classification of Diseases and Related Health Problems-10 (ICD-10) codes X00-X09], and was considered a home death if the place of injury was coded “home”. US population data produced by the US Census Bureau and NCHS were obtained from the Centers for Disease Control and Prevention Wide-ranging Online Data for Epidemiologic Research (CDC WONDER 2013).

Covariates of interest were sex (female, male), age (in years, 0–14, 15–64, 65 or older), ethnicity (Hispanic, non-Hispanic), race (Black, White, other), and state of death (fifty states of the US and DC). These covariates were available on the mortality multiple cause-of-death data and US population data.

### Implementation of FSC Legislation

We obtained data on the effective date of FSC legislation for all 50 states and DC from the website of the Coalition for Fire-Safe Cigarettes, a group coordinated by the National Fire Protection Agency. The intervention assessed was the effective date of each state’s FSC legislation (Coalition for Fire-Safe Cigarettes 2011). Since the death data did not contain day of death, we classified FSC legislation by month of implementation. If FSC legislation was implemented for at least half of a month, then the entire month was considered to be under FSC legislation implementation. If less than half of a month was under FSC legislation implementation, then the entire month was considered to be not under FSC legislation implementation.

### Statistical analysis

We used a Poisson regression analysis of national mortality data to quantify the association between state-level implementation of FSC legislation and the rate of residential fire mortality, by state and month. Unadjusted rate ratios (uRRs) and adjusted rate ratios (aRRs), along with their Wald-based 95% confidence intervals (95% CIs) and p-values, were calculated. To account for possible delayed effects of law implementation and residential fire death, we created 12-month and 24-month lag function. We also conducted subgroup analyses by demographic variables and by state. As per NCHS guidance, we did not present results for subgroup analyses where any strata had fewer than 10 deaths.

The population at risk was assumed to be constant within each state in each calendar year. All adjusted analyses control for changes in age, gender, ethnicity, race between states, and over time within each state. We also included state as a categorical variable in all models in order to adjust for between-state differences in the rate of residential fire mortality (e.g., due to geographical differences in house construction and household heating). All data analysis was performed with SAS 9.3 (Cary, NC).

## Results

From 2000–2010, there were 29,910 residential fire-related deaths in the US. A total of 184 deaths (0.6%) had either age or ethnicity of the decedent unknown. Since the proportion of missing demographic data was low, we elected to restrict analysis to those with complete data, resulting in a final sample of 29,726 fire-related deaths occurring in the home. The overall death rate from 2000–2010 was 9.14 per 1,000,000 person-years (Table 1). Males had a higher fire-related death rate than females (10.67 vs. 7.66 per 1,000,000 person-years, respectively), and older adults (65+ years) had a higher death rate (25.25 per 1,000,000 person-years) than other age groups (0–14 years and 15-64-years). In terms of ethnicity and race, non-Hispanic people had a higher fire-related death rate than Hispanic people (10.03 vs. 3.91 per 1,000,000 person-years, respectively) and Black people had the highest death rate (16.85 per 1,000,000 person-years).

**Table 1:** **Residential fire deaths, 2000–2010**

	N	Person-years	Rate*	95% CI	uRR	95% CI
Total	29,726	3,252,993,222	9.14	9.03, 9.24		
Sex						
Male	17,048	1,598,125,310	10.67	10.51, 10.83		ref
Female	12,678	1,654,867,912	7.66	7.53, 7.79	0.72	0.70, 0.73
Age						
0–14 years	4,668	667,491,362	6.99	6.79, 7.19		ref
15–64 years	14,743	2,177,035,230	6.77	6.66, 6.88	0.97	0.94, 1.00
65+ years	10,315	408,466,630	25.25	24.77, 25.74	3.61	3.49, 3.74
Ethnicity						
Non-Hispanic	27,869	2,778,280,286	10.03	9.91, 10.15		ref
Hispanic	1,857	474,712,936	3.91	3.73, 4.09	0.39	0.37, 0.41
Race						
White	21,688	2,620,757,879	8.28	8.17, 8.39		ref
Black	7,299	433,145,138	16.85	16.46, 17.24	2.04	1.98, 2.09
Other	739	199,090,205	3.71	3.44, 3.98	0.45	0.42, 0.48

*crude rate per 1000000 person-years.

*Abbreviations:* CI = confidence interval, ref = reference, uRR = unadjusted rate ratio.

Excludes 184 deaths where age or ethnicity are unknown.

Unadjusted Poisson regression models indicated that the residential fire death rate was 31% lower after implementation of FSC legislation, compared to prior to implementation of FSC legislation [uRR (95% CI) = 0.69 (0.67, 0.72)]. The protective effect of legislation persisted even after adjusting for covariates (Table 2). The covariate-adjusted residential fire death rate was 19% lower following implementation of FSC legislation [aRR (95% CI) = 0.81 (0.79, 0.84)]. There was no evidence effect of delayed effects of law implementation in any of the lag models.

**Table 2:** **Overall and stratum-specific rate ratios (post-FSC law vs. pre-law) associated with residential fire deaths, 2000–2010**

	RR	95% CI	***p***-value
*Overall models*			
Unadjusted model			
Fire-safe cigarette implementation	0.69	0.67, 0.72	<0.0001
Adjusted model*			
Fire-safe cigarette implementation	0.81	0.79, 0.84	<0.0001
*Stratum-specific models*			
Sex			
Male	0.67	0.65, 0.70	<0.0001
Female	0.72	0.67, 0.76	<0.0001
Age			
0–14 years	0.52	0.48, 0.57	<0.0001
15–64 years	0.69	0.66, 0.73	<0.0001
65+ years	0.77	0.73, 0.81	<0.0001
Ethnicity			
Non-Hispanic	0.72	0.70, 0.75	<0.0001
Hispanic	0.71	0.64, 0.80	<0.0001
Race			
White	0.75	0.73, 0.78	<0.0001
Black	0.59	0.55, 0.63	<0.0001
Other	0.62	0.52, 0.74	<0.0001

*Adjusted for age, sex, ethnicity, race, and state of death.

*Abbreviations:* CI = confidence interval, RR = rate ratio.

We also conducted unadjusted sub-analyses within strata of age, sex, race, ethnicity, and state. FSC implementation was associated with a protective effect in every demographic stratum (Table 2). We limited analysis of state-specific effects to the 44 jurisdictions with 10 or more deaths in both the pre-implementation and post- implementation periods. In 32 of the 44 jurisdictions, residential fire mortality rates decreased by at least 5% (Table 3). In 12 jurisdictions there was either a decrease of less than 5%, or else the rate increased following implementation of FSC legislation.

**Table 3:** **FSC legislation implementation and residential fire mortality rates, by state (and District of Columbia), 2000–2010**

		Before state law effective			After state law effective		
	Law effective	Deaths	Person-years	Death rate*	Deaths	Person-years	Death rate*
New York	6/28/2004	688	85,984,141	8.00	843	124,870,068	6.75
Vermont	5/1/2006	47	3,903,907	12.04	21	2,913,619	7.21
California	1/1/2007	1171	246,016,158	4.76	628	147,154,075	4.27
Oregon	7/1/2007	213	26,673,202	7.99	79	13,276,889	5.95
New Hampshire	10/1/2007	70	9,925,735	7.05	34	4,276,950	7.95
Illinois	1/1/2008	921	100,543,665	9.16	231	38,385,796	6.02
Maine	1/1/2008	74	10,448,371	7.08	25	3,987,478	6.27
Massachusetts	1/1/2008	326	51,255,723	6.36	82	19,542,046	4.20
Kentucky	4/1/2008	532	34,201,422	15.55	157	11,881,706	13.21
Montana	5/1/2008	51	7,755,012	6.58	19	2,625,883	7.24
New Jersey	6/1/2008	387	72,333,057	5.35	110	22,636,664	4.86
Connecticut	7/1/2008	193	29,608,267	6.52	51	8,910,095	5.72
District of Columbia	7/1/2008	107	4,858,303	22.02	37	1,487,258	24.88
Maryland	7/1/2008	412	46,884,955	8.79	116	14,358,552	8.08
Utah	7/1/2008	98	20,527,236	4.77	28	6,830,415	4.10
Alaska	8/1/2008	87	5,635,277	15.44	15	1,699,481	8.83
Rhode Island	8/1/2008	67	9,123,071	7.34	22	2,545,759	8.64
Minnesota	12/1/2008	259	45,376,831	5.71	61	11,029,113	5.53
Delaware	1/1/2009	63	7,497,088	8.40	14	1,791,522	7.81
Iowa	1/1/2009	250	26,653,976	9.38	53	6,083,072	8.71
Oklahoma	1/1/2009	522	31,886,682	16.37	125	7,477,756	16.72
Pennsylvania	1/1/2009	1406	111,836,575	12.57	254	25,384,580	10.01
Texas	1/1/2009	1816	202,658,125	8.96	333	50,055,227	6.65
Idaho	4/1/2009	83	13,039,891	6.37	23	2,736,931	8.40
Indiana	7/1/2009	684	59,450,591	11.51	90	9,720,285	9.26
Kansas	7/1/2009	311	26,083,198	11.92	39	4,275,495	9.12
West Virginia	7/10/2009	236	17,289,303	13.65	37	2,778,256	13.32
Colorado	7/31/2009	182	44,293,115	4.11	34	7,119,440	4.78
Arizona	8/1/2009	337	55,009,520	6.13	37	9,056,139	4.09
Washington	8/1/2009	433	59,772,551	7.24	71	9,521,044	7.46
Louisiana	8/31/2009	793	43,205,199	18.35	80	6,042,559	13.24
Hawaii	9/30/2009	29	12,464,189	2.33	^	1,700,038	^
Wisconsin	10/1/2009	446	53,846,765	8.28	34	7,108,975	4.78
Alabama	1/1/2010	714	45,781,886	15.60	74	4,785,401	15.46
Arkansas	1/1/2010	569	27,773,493	20.49	33	2,921,588	11.30
Florida	1/1/2010	1154	175,070,073	6.59	103	18,838,613	5.47
Georgia	1/1/2010	1286	89,062,054	14.44	112	9,712,157	11.53
Michigan	1/1/2010	1161	99,992,729	11.61	88	9,877,143	8.91
Nebraska	1/1/2010	155	17,576,652	8.82	14	1,830,141	7.65
New Mexico	1/1/2010	136	19,221,530	7.08	^	2,065,913	^
North Carolina	1/1/2010	1049	87,093,319	12.04	52	9,560,234	5.44
South Carolina	1/1/2010	640	42,749,206	14.97	46	4,637,106	9.92
Tennessee	1/1/2010	1090	59,818,027	18.22	102	6,357,436	16.04
Virginia	1/1/2010	740	75,194,867	9.84	68	8,023,953	8.47
Ohio	5/1/2010	1140	118,381,152	9.63	81	7,691,979	10.53
Nevada	6/1/2010	154	24,906,956	6.18	^	1,577,498	^
Mississippi	7/1/2010	754	30,448,237	24.76	30	1,485,036	20.20
North Dakota	8/1/2010	52	6,867,179	7.57	^	281,095	^
Missouri	1/1/2011	915	63,781,731	14.35	N/A	N/A	N/A
South Dakota	1/1/2011	88	8,580,899	10.26	N/A	N/A	N/A
Wyoming	7/1/2011	33	5,743,681	5.75	N/A	N/A	N/A

*Rate per 1,000,000 person-years.

^Numbers not displayed due to counts <10.

*Abbreviation:* N/A = not applicable.

## Discussion

Residential fire death rates in the 50 states and DC were lower following implementation of FSC legislation. Smoking materials accounted for 23% of home fire deaths in 2010, thus it is not realistic to see a 100% reduction in residential fire deaths as a result of FSC legislation. This estimated reduction reflects a substantial cost savings in the US, as each home fire-related death results in medical and work loss cost of over $750,000 (National Center for Injury Prevention and Control 2013). FSC legislation has also been passed outside the US in locations including Australia, Canada, and the European Union (Australian Competition and Consumer Commission 2014; European Commission 2011; Health Canada 2012); to our knowledge no research has been conducted on the association of FSC legislation and fire deaths in these other locations.

We observed some variations in the effect of FSC laws between states. These variations could be related to nuances of the passage and implementation of the law between states. However, they may also reflect simple random variation in the number of fire-related deaths before and after the implementation of FSC legislation in each state. A longer time series and detailed data on the state-level implementation (such as enforcement and testing resources) is required to definitively assess state effects.

A protective effect for FSC legislation was present in every demographic subgroup examined (Table 2). The protective effect appeared to be stronger in children (0–14 years) and non-Whites (Table 2). The reasons for these variations are unclear but may be related to the variation in the proportion of residential fire deaths that are smoking-related across demographic subgroups.

Some caution is needed in the interpretation of the results. First and foremost, standard sources of mortality data do not permit disaggregation by ignition source. Thus, there is no simple means to quantify whether changes in the rate of residential death were due to declines in smoking materials as ignition sources using national mortality data. The observed decline of 19% is approximately consistent with proportion of residential fire deaths (23%) in which smoking materials are an ignition source.

Second, a variety of factors external to implementation of FSC legislation could have led to the decrease in residential fire deaths, including decreases in cigarette consumption, creation of flammability standards for mattresses and upholstered furniture, changes in home construction, increases in residential smoke detectors and sprinkler systems, and changes in proportion of US households that prohibit indoor smoking (Hall Jr. 2013). For example, from 1980 to 2006, cigarette consumption decreased by 41% (Hall Jr. 2013). Additional changes in other risk factors associated with residential fire death, such as improvements in home construction, the introduction of fire alarm and sprinkler systems, (Baker et al. 1992; Marshall et al. 1998; Runyan et al. 1992), and improvements in trauma systems and burn and critical care, could also have contributed to the observed 19% decrease. We also lack data on sales of cigarette across state borders or via the internet. It would require substantial resources to assemble a complete set of data on all these covariates for all states for all the years included in this study.

Third, national mortality data were available only through 2010, and over half (33/51) of the states implemented FSC legislation during or after 2009, thus there was a limited amount of state-years in this study following FSC legislation implementation. The limited amount of state-years in this study hampers our assessment of delayed effects of FSC legislation using lag models. Finally, this study only addressed fire-related mortality; there is no reliable national morbidity data that would permit state-level estimates. However, data from the National Fire Incident Reporting System (a volunteer reporting system for fire response agencies) indicates that non-fatal fires with smoking materials as an ignition source also declined over the study period (from 900 to 540 reported annually) (National Fire Protection Agency, personal correspondence). Interventions designed to reduce injuries can be classified as active or passive, where passive interventions “do not require the continued, active cooperation of the public” (Haddon Jr. 1980). Passive interventions are typically considered to be more efficacious than active interventions (Haddon Jr. 1980). FSC legislation, a passive intervention, can therefore be expected to be highly successful. As noted, the benefits of the intervention appear to be substantial and the intervention generates no harms, other than cost of testing cigarettes to ensure compliance.

## Conclusions

In adjusted analyses, implementation of FSC legislation was associated with a 19% decrease in the rate of fire-related deaths in the home. However, this study cannot definitely demonstrate a causal relationship, in part due to limitations in the available data. Future research on this topic would benefit from enhancements to national surveillance data systems to capture information on ignition source for national mortality and nationally-representative morbidity data.

## Authors’ information

RKY is a doctoral student in injury epidemiology at the University of North Carolina at Chapel Hill. SWM is a professor of epidemiology at the University of North Carolina at Chapel Hill and Director of the University of North Carolina Injury Prevention Research Center, which is funded in part by the Centers for Disease Control and Prevention.

## Abbreviations

aRR: Adjusted rate ratio
CI: Confidence interval
DC: District of Columbia
FSC: Fire-safe cigarette
ICD-10: International Statistical Classification of Diseases and Related Health Problems-10
NCHS: National Center of Health Statistics
uRR: Unadjusted rate ratio
US: United States.
